# Untargeted Metabolomics Reveals Functional Bioactive Metabolite Networks in Green Rice Milk Yogurt Enriched with *Wolffia globosa*

**DOI:** 10.3390/life16081385

**Published:** 2026-08-21

**Authors:** Pongpat Kiatprasert, Sukrita Punyauppa-Path, Nonthiwat Taesuk, Sujira Maneerat, Watchara Kanchanarach, Priyapa Najomtien, Srisan Phupaboon

**Affiliations:** 1 Department of Mathematics and Science, Faculty of Agriculture and Technology, Rajamangala University of Technology Isan Surin Campus, Surin 32000, Thailand; pongpat.ki@rmuti.ac.th (P.K.); phira.bo@rmuti.ac.th (S.P.-P.); 2 Department of Biology, Faculty of Science, Mahasarakham University, Mahasarakham 44150, Thailand; nonthiwat.t@msu.ac.th (N.T.); sujira.m@msu.ac.th (S.M.); priyapa.n@msu.ac.th (P.N.); 3 Faculty of Public Health, Mahasarakham University, Mahasarakham 44150, Thailand; watchara.k@msu.ac.th

**Keywords:** bio-fermentation, metabolomics network, bioactive-mapping pathway, superfoods, plant-based yogurt, functional foods

## Abstract

This study aimed to illustrate the effects of plant-based yogurts made from bio-fermentative green rice milk yogurt (GRMY), enhanced with *Wolffia globosa* powder (WGP) and infused with Thai green tea. Different formulations (F1 to F4) were analyzed to assess the impact of varying weight ratios of WGP, ranging from 0% to 15%. The goal was to improve nutritional values and bioactive components. The F4 formulation of plant-based yogurt demonstrated enhanced nutritional values, including higher levels of dietary fiber, starch, protein, and fat contents, which were 42.45, 26.55, 22.48, and 2.29% DW, respectively. It was increased by bioactive compounds such as total phenolic content (52.99 mg GAE/g), total flavonoid content (60.45 mg QUE/g), and β-carotene (1.81 mg/g). Additionally, the F4 formulation exhibited the highest antioxidants in terms of DPPH activity (5.48 mg TROE/g), ABTS activity (424.15 mg TROE/g), and FRAP reducing capacity (106.35 mg TROE/g) and antidiabetic activity of α–glucosidase inhibition in IC_50_ at 1.41 mg/mL. The yogurts were evaluated through metabolomic network analysis as a complex bioactive food system, wherein fermentation-derived metabolites synergistically interact with tea polyphenols, amino acids, phytosterols, and carotenoids. The prevailing metabolic profiles indicate an increase in antioxidant capacity, a reduction in inflammatory burden, enhanced glucose metabolism, alterations in gut microbiota, cardiovascular protection, and neuroprotective potential. These findings suggest that the product represents a promising plant-based functional food, offering numerous health benefits. This assertion is supported by a diverse metabolite network identified through UPLC–MS/MS metabolomic research.

## 1. Introduction

In recent years, consumer concerns regarding the nutritional and health benefits of food have escalated, leading to a heightened need for non-dairy milk alternatives (plant-based milk) that are widely accepted and useful in diets [1]. Plant-based milk is transitioning from a niche alternative to a mainstream dietary option, rich in vitamin B, characterized by its significant market expansion, product innovation (beyond mere animal substitutes), and various offerings, sometimes known as vegan or non-dairy yogurts [2,3]. The FDA has released draft guidance regarding the labeling of plant-based substitutes for animal-derived products, including plant-based yogurts [4]. Those advertised and sold as milk replacements are defined as those derived from nuts (e.g., hazelnuts, walnuts, cashews, almonds), seeds (such as sesame and flax), white rice, oats, or legumes, including soy [5]. The FAO is the mandated United Nations custodial agency for 21 SDG indicators and the collaborating agency for five more, including functional foods that make a difference in healthy consumption [6], covering more than 10% of the total SDG global indicator framework. The best solution for this research, based on consumer needs, is functional foods to significantly improve health, which contain potentially beneficial functions and biological compounds, especially rice/brown rice-based milk [7,8], riceberry rice milk [9,10], and watermeal [11].

Thai rice (*Oryza sativa*), Hom Mali and/or Khao Dawk Mali (105), has low allergenic potential; so, it is suitable for people with lactose intolerance or those allergic to nuts or soy. Hom Mali is defined by its elevated glycemic content and its abundant nutritional profile, which includes dietary fiber, vitamins, minerals, and antioxidant properties [12,13]. Other phytochemical compositions had increased α-amylase activity, reduced sugars, crude protein, free amino acids and total phenolic content. Protein enrichment was associated with increases in essential and non-essential amino acids, especially branched-chain amino acids, alanine, glycine and proline. In addition, B-group vitamins (B1, B2, B3 and B6) increased after germination [14,15]. The metabolites discussed include GABA, a non-protein amino acid that functions as an inhibitory neurotransmitter in the brain. Benefits linked to GABA consumption encompass stress reduction, improved sleep quality, potential hypertension prevention, cancer cell proliferation inhibition, and blood pressure management relevant to anti-type 2 diabetes [10]. Watermeal (*W. globosa*) is a superfood source of nutritional profiles, protein (up to 41%), starch, dietary fiber, polyunsaturated fatty acids, essential amino acids, and various bioactive compounds (e.g., TPC, TFC, chlorophyll, and β-glucan contents) [16,17,18].

Additionally, yogurt is a fermentation product of lactic acid bacteria (LAB), rich in proteins, polysaccharides and other nutrients [19,20]. During fermentation, the proteins in yogurt are hydrolyzed to amino acids, and the polysaccharides are hydrolyzed to monosaccharides, thereby facilitating digestion and absorption [21]. This product is a complex food matrix from a molecular point of view, consisting of a variety of biomolecules such as lipids, carbohydrates, proteins and many small metabolites including organic acids, fatty acids, nucleic acids, amino acids, aroma volatiles and minerals, which together define the unique flavor profile of yogurt [22]. Metabolomics enables complete analysis of chemical composition, providing qualitative and quantitative information on thousands of metabolites, including those that are normally ignored by classical approaches. The changes in these metabolites directly influence the quality and nutritional properties of foods [23,24], leading to their applications in food and nutrition research.

Hence, the present study demonstrated that GRM with variable ratios of WGP supplementation produces plant-based yogurts exhibiting a bio-fermentation process. The study aimed to evaluate the contribution of biological activity and bionutritional traits to the bioactive-based metabolomic network pathways, based on their phytochemical compounds and end products through untargeted LC-MS analysis.

## 2. Materials and Methods

### 2.1. Rice Raw Materials and Microorganism Inoculums

This study evaluates green rice milk (GRM), scientific name *O. sativa* L. (Khao Dawk Mali 105), also locally known as Khao Mao milk, produced from rice harvested before full maturity (30 days post-flowering), and *W. globosa* powder (WGP) and Thai green tea powder, sourced from the previous study of Phupaboon et al. [16], as a plant-based protein, fragrant, and phytonutrient source. All ingredients were purchased from a local market in Khon Kaen and Mahasarakham provinces, Thailand. Furthermore, the commercial microorganisms *Lactobacillus delbrueckii* subsp. *bulgaricus* and *Streptococcus thermophilus* were procured from Dairy LB81-Bulgaria Yogurt (CP-Meiji, Bangkok, Thailand). Additional organic solvents and reagents were obtained from Sigma-Aldrich (Sigma-Aldrich™, Burlington, MA, USA).

### 2.2. Production of GRMY-WGP

**Figure 1:**
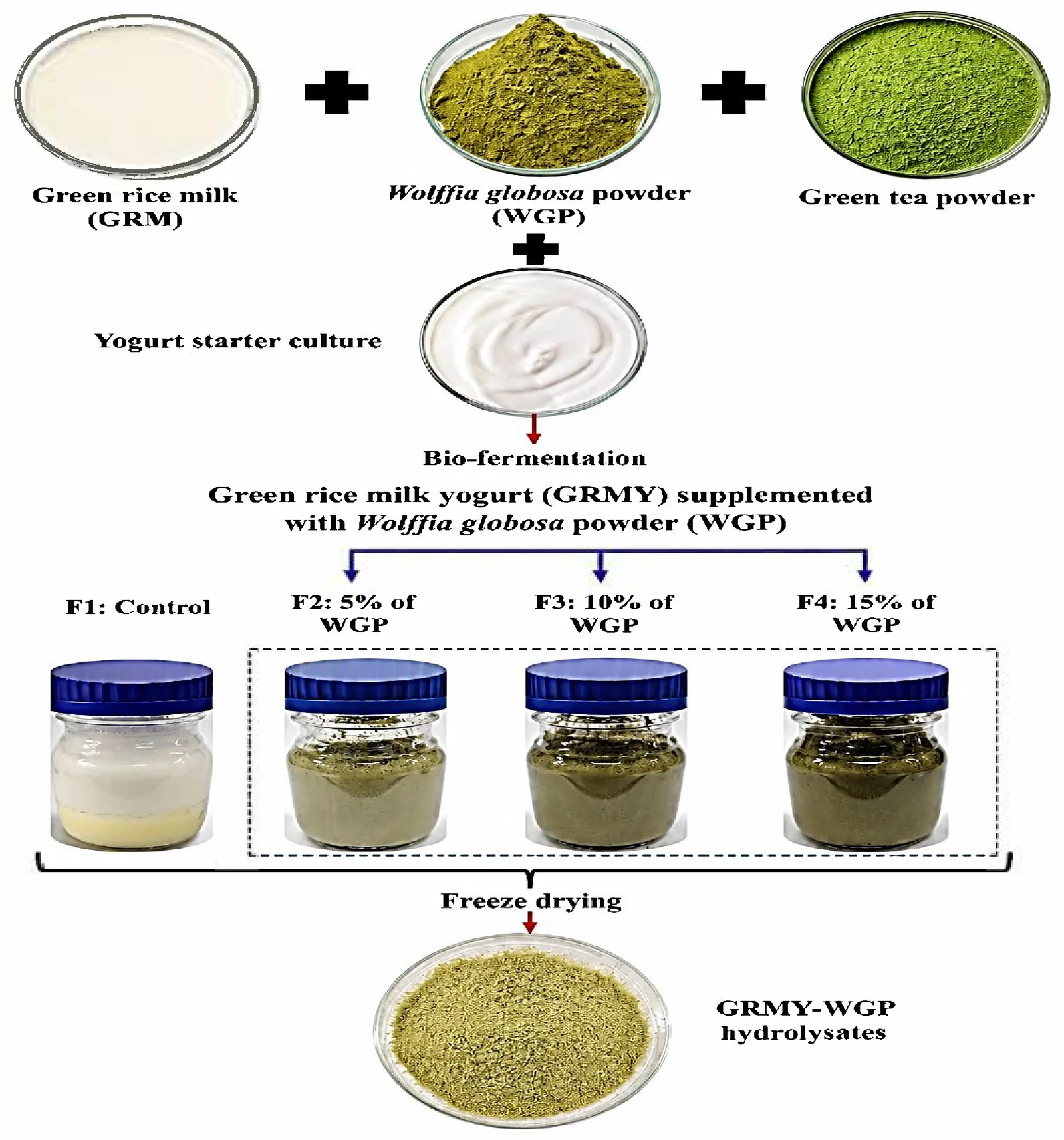
The procedure for producing GRMY-WGP and its formulations.

The green rice milk yogurt enriched with *W. globosa* powder (GRMY-WGP) formulations were prepared by modifying the method previously described by [16]. Briefly, sterilized GRM was supplemented with WGP at four different levels (0, 5, 10, and 15%, *w*/*w*), designated as F1, F2, F3, and F4, respectively. Thai green tea powder was added at a constant concentration to all formulations to provide a characteristic aroma and flavor. The mixtures were inoculated with a commercial yogurt starter culture at 2% (*w*/*w*, based on the total formulation) and incubated under controlled fermentation conditions as described below. Following fermentation, the samples were immediately cooled to terminate the fermentation process and stored at 4 °C until subsequent physicochemical, bioactivity, and metabolomic analyses. The detailed formulation of each treatment is presented in Figure 1.

### 2.3. Determination of Nutritive Values

The proximate analysis of GRMY-WGP hydrolysates was performed following AOAC protocols [25], including crude fat, crude protein, carbohydrate, and crude fiber contents. All experiments were done in triplicate and were reported in (%, DW).

### 2.4. Determination of Phytochemical Compositions

The phytochemical profiles of GRMY-WGP hydrolysates were analyzed using spectrophotometry methods. The total phenolic content (TPC) was determined using Folin–Ciocalteu reagent as mg Gallic acid equivalent (GAE)/g dry sample at 765 nm. The total flavonoid content (TFC) was measured using 10% aluminum chloride solution and expressed as mg Quercetin equivalent (QUE)/g dry sample at 415 nm. DPPH, ABTS, and FRAP assays at 517, 734, and 593 nm, respectively, were used to determine the antioxidant activity (AA), when compared with mg Trolox equivalent (TROE)/g dry sample [26,27]. In addition, β-carotene (mg/g) was measured by the acetone extraction method and read at 449 nm using a UV–Vis spectrophotometer [28]. All analyses were done in triplicate.

### 2.5. Determination of Antidiabetic Activity

The α-glucosidase inhibitory activity was determined using a 96-well microplate assay. Each reaction mixture contained α-glucosidase (0.1 U/mL), 4-nitrophenyl-α-D-glucopyranoside (pNPG) as the substrate, and serially diluted sample solutions prepared from a 10 mg/mL stock solution. The absorbance was measured at 405 nm before incubation (0 min) and after incubation at 37 °C for 15 min using a microplate reader [29]. The percentage inhibition of α-glucosidase activity was calculated at each sample concentration relative to the acarbose positive control. Dose–response curves were constructed by nonlinear regression analysis, and the inhibitory concentration required to inhibit 50% of enzyme activity (IC_50_) was expressed as mg/mL.

### 2.6. Metabolomics Analysis of GRMY-WGP Hydrolysates

The metabolomic profiles of GRMY–WGP were characterized using UPLC–QTOF–MS/MS following the analytical procedure described by [16]. Based on the bioactivity results, the F1 (0% WGP) and F4 (15% WGP) formulations were selected for comparative metabolomic analysis. Freeze-dried samples were dissolved in the extraction solvent to obtain a final concentration of 20 mg/mL, filtered through a 0.22 μm syringe membrane filter, and subjected to UPLC–QTOF–MS/MS analysis. Chromatographic separation was performed using a CORTECS C18 column (2.1 × 100 mm, 1.6 μm) coupled with a CORTECS T3 VanGuard pre-column (2.1 × 5 mm, 1.6 μm) (Waters Pacific Pte. Ltd., Drinagh, Ireland). Electrospray ionization (ESI) was operated in both positive and negative ionization modes with capillary voltages of 2.5 and 2.3 kV, respectively. A sample injection volume of 3 μL was used for each analysis. Gradient elution was carried out using mobile phase A (0.1% formic acid in water) and mobile phase B (acetonitrile) at a flow rate of 0.4 mL/min, while the column temperature was maintained at 30 °C. The total chromatographic run time was 28.1 min.

The acquired UPLC–QTOF–MS/MS data were processed using Progenesis QI and annotated against the METLIN (version 3.0) database. The resulting metabolite dataset was subsequently exported to EZinfo (version 3.1), while integrative metabolomics analysis and biochemical pathway visualization were performed using MetaboAnalyst (version 4.0) and MetaMapp (accessed on 16 August 2026) [30,31]. The reported compounds represent putatively annotated metabolites based on accurate mass matching and available database information and should not be interpreted as unequivocally identified metabolites.

### 2.7. Statistical Analysis

All experiment statistics were presented as the mean ± SD values from (*n* = 3). The variances between treatments were tested using the analysis of variance (ANOVA) by Duncan’s new multiple range test (*p* < 0.05) using IBM SPSS-KKU Statistics v27.0 software. Additionally, the UPLC-QTOF data was uploaded to Progenesis QI in conjunction with the METLIN v3.0 database, resulting in the identification of untargeted small molecules. The identified compounds were subsequently exported to EZ info v3.1. Additionally, we conducted integrative metabolomics data analysis using MetaboAnalyst v4.0 and integrated biochemical pathways using MetaMapp (accessed on 16 August 2026) [30,31].

## 3. Results and Discussion

### 3.1. Fermentation Characteristics and Nutritive Values of GRMY-WGP Hydrolysates

As mentioned in a previous study, the created *Wolffia*-based alternative protein yogurt, particularly the WSbY-F4 formulation, showed optimal fermentation properties. After 8 h of fermentation, it achieved the highest pH (4.45), lactic acid bacterial count (9.3 log CFU/mL), total soluble protein level (260 mg/g), and trichloroacetic acid-soluble peptides (120 mg/g), indicating improved bio-nutrition, bio-fermentation, and protein digestibility [16]. The current results of the nutritional contents of several GRMY-WGP hydrolysates, specifically the F1, F2, F3, and F4 formulas, indicate a significant difference (*p* < 0.05) in the F4 yogurt hydrolysate containing 15% WGP when compared to the other formulations, as presented in Table 1. Each GRMY-WGP hydrolysate exhibited protein contents ranging from 16.68% to 22.48% DW, starch contents between 10.58% and 26.55% DW, dietary fiber contents from 7.59% to 42.45% DW, and fat contents from 0.38% to 2.29% DW, respectively. A similar result of rice milk (*O. sativa*) in Sri Lanka showed a high proximate composition in terms of carbohydrate, protein, and dietary fiber contents and a lower content of fat [32]. In addition, the study of da Silva et al. [33] characterized three different types of rice (red, black, and white)-based milk products obtained from pasteurization; there was a significant difference in the carbohydrate, protein, and lipid contents of red, black, and white rice. In contrast to cow milk (3.7%), almond milk (2.11%), and soy milk (3.70%), rice milk possesses a negligible protein content (1.83%), but rice milk is rich in the glycemic index (GI) [8]. GI measures the rate at which carbohydrates are converted into sugar after consumption. This is crucial for controlling blood sugar levels, preventing diabetes, managing weight, and maintaining the body’s energy balance each day [34]. There is also a lower fat content (<1%) found in rice milk; bovine milk is high in saturated fatty acids with about 2.5 g/100 g, whereas plants generally have a low content. This research has led to the invention and development of a plant-based yogurt formula rich in protein, such as *W. globosa* powder (WGP), an aquatic plant with a high protein content, to enhance nutritional value, especially the necessary protein intake to meet the required levels [16]. For example, for newborns, consuming nutritious food appropriate to their needs is crucial, as approximately 40% of protein is needed for growth. Adults also need protein for maintenance; therefore, the protein requirement in grams/kilogram/day is lower. Currently, the latest FAO report indicates that the protein requirement for adults is 0.66 g/kg/day, and for infants aged 0.5 years, it is 1.12 g/kg/day [35].

### 3.2. Phytochemical Capacities of GRMY-WGP Hydrolysates

In addition, the biological activities including TPC, TFC, and AA in terms of the radical scavenging activity inhibitions, DPPH, ABTS, and FRAP capacity, as well as their antidiabetic activity such as α–glucosidase activity inhibition obtained from GRMY-WGP hydrolysates are represented in Table 2. The present findings indicated that the TPC of all hydrolysates varied from 22.06 to 52.99 mg GAE/g DW. The TFC varied between 24.97 and 60.45 mg QUE/g DW. The antioxidant-based reducing capabilities, measured by DPPH, ABTS, and FRAP, ranged from 3.82 to 5.48 mg TROE/g DW, 340.29 to 424.15 mg TROE/g DW, and 29.18 to 106.35 mg TROE/g DW, respectively. The β-carotene concentration ranged from 0.69 to 1.81 mg/g DW. The antidiabetic action of the four hydrolysates, F1 to F4, exhibited IC_50_ values of 1.73, 1.53, 1.47, and 1.41 mg/mL for α-glucosidase activity inhibition. In particular, the F4 yogurt hydrolysates exhibited markedly elevated levels of phytochemical substances and biological activity compared to the other groups. The most notable outcome from this data is the emergence of new information regarding a small database of the prospective application of GRMY augmented with a WGP-based protein supply. Numerous studies have examined rice-based milk proteins (germinated brown rice, and red rice milk), as well as green rice or Khao Dawk Mali 105 (30 days post-flowering), and its milling fractions, which include whole grain green rice, broken green rice, white broken green rice, and green rice bran, focusing on their bioactive components, antioxidant properties, prebiotic oligosaccharides, and amino acid profiles [7,15]. Riceberry rice is abundant in anthocyanins, possesses a low glycemic index, and demonstrates significant antioxidant capacity, hence exhibiting superior nutritional qualities and bioactive components; it can influence human health regarding its antioxidant and antidiabetic effects [9]. Alternative sources of nutrition and bioactive compounds come from *W. globosa*, which exhibited the highest quantities of protein (22.7%), dietary fiber (16.5%), TFC (5.0 mg QE/g DW), and TPC (3.9 mg GAE/g DW). Other concentrations of chlorophyll and β-glucan demonstrated the highest levels of total amino acids [11,36]. Hu et al. [37] conducted another investigation indicating that the high-protein strain of *Wolffia arrhiza* possesses a protein content of 50.89%, implying that the protein is of superior quality. They comprise 89.7% total fatty acids, with 70.85% classified as unsaturated fatty acids, specifically palmitic, linoleic, and α-linolenic acids. Prior research on the antidiabetic potential of riceberry rice yogurt, which examined the immediate impact on plasma glucose levels post-consumption, originates from a study that reported a rapid enhancement in the plasma FRAP, TEAC, and ORAC [9]. Yogurt containing BCP demonstrated the most significant α-glucosidase inhibition (>90%) with an IC_50_ of 0.20 mg/mL, and the enhancement in bioactivity is ascribed to the vitality of lactic acid bacteria, sustained at roughly 10^8^ CFU/g [38]. Recent evidence of Phupaboon et al. [16] proposed an innovative RBRM-based yogurt formulated with 15% WGP (F4 formulation), which demonstrated superior nutritional contents, antioxidant capacity, and antidiabetic activity. Twelve constituents of the top metabolites exhibited elevated quantities of the released biological compounds.

### 3.3. Untargeted Metabolites of GRMY-WGP Hydrolysates-Based Biological Compounds

The analysis of UPLC-QTOF MS/MS data derived from control (F1) and the GRMY-WGP containing 15% *Wolffia* powder in yogurt hydrolysate forms (F4) yielded the identification of 1009 structures in negative mode and 987 structures in positive mode (Figures S1 and S2). The untargeted UHPLC–QTOF–MS/MS workflow generated numerous metabolic features. Following background correction, feature filtering, and metabolite annotation, 88 putatively annotated metabolites were retained for subsequent analyses (Figures S1 and S2). These metabolites are presented in Supplementary Table S1. In addition, among these, 48 representative metabolites with relatively high biological relevance were selected from the 88 putatively annotated metabolites for functional classification and biological interpretation (Supplementary Table S2). Twelve key metabolites were subsequently highlighted for detailed discussion because of their reported bioactivities and relevance to the objectives of the present study. The top metabolites-based bioactive compounds from GRMY-WGP were categorized in green rice and/or green tea-related metabolites (e.g., L-theanine, gallocatechin, rutin, and quercetin derivatives), fermentation metabolites (e.g., lactobionic acid, organic acids, and amino acids), and functional metabolites (e.g., kaempferol glycosides, phenolic acids, and azelaic acid), as presented in Table S2. The metabolites discussed below represent putatively annotated metabolites selected for biological interpretation. Their annotations were assigned based on database matching and should not be regarded as unequivocally identified compounds. Confirmation using authentic standards and MS/MS spectral matching is required for definitive metabolite identification. Metabolites lacking sufficient biological plausibility or annotation confidence were excluded from further biological discussion. In accordance with our previous companion study [23], nine representative metabolites, namely leucodelphinidin 3-O-α-L-rhamnopyranoside, macaflavone II, vignatic acid B, dihydroresveratrol, embelin, nateglinide, acitretin, mangostenone B, and indoramin, were selected from riceberry rice milk yogurt supplemented with *W. globosa* powder (RBRMY-WGP) hydrolysates analyzed by UPLC–QTOF–MS/MS. These metabolites are presented here solely as literature-derived representative metabolites to facilitate biological interpretation and comparison and should not be interpreted as metabolites experimentally identified in the present of GRMY-WGP metabolomic dataset. This study’s findings also align with prior research, revealing that black rice contains phytonutrients identified through targeted LC-MS/MS analysis, including oryzanol, ferulic acid, 4-hydroxybenzoic acid, apigenin, tricin, avenasterol, coumarin, coumaric acid, phenylalanine, caffeic acid, α-tocopherol, protocatechuic acid, and dehydroxy myricetin [39]. Additional chemicals with significant anticancer action were identified in Thai riceberry rice, including anthocyanins, cyanidin-3-glucoside, and peonidin-3-glucoside, as determined by GC–MS and LC–ESI–MS/MS [40]. Other studies reported the different sources of plant-based protein yogurts: almond, soybean, coconut, and oat products, which showed various metabolite and key metabolomic pathways, including amino acids and derivatives, saccharides, organic acids, and flavonoids, among others [1,20,41]. Surveys such as that conducted by Yu et al. [42] have shown that the metabolic pathways using GC-GC-TOF-MS- and LC-ESI-MS/MS-based flavoromics and metabolomics recognized for both soy-based and dairy-based samples encompass pyruvate and glycolysis/gluconeogenesis metabolism among volatile or non-volatile metabolites. In accordance with the previous result of Lee et al. [43], integrating the investigation of volatile and non-volatile metabolites offers a thorough understanding of LAB fermentation processes, particularly *Latilactobacillus* sp.

### 3.4. Bioactive Metabolite Network of GRMY-WGP: UPLC–MS/MS-Based Interpretation

**Figure 2:**
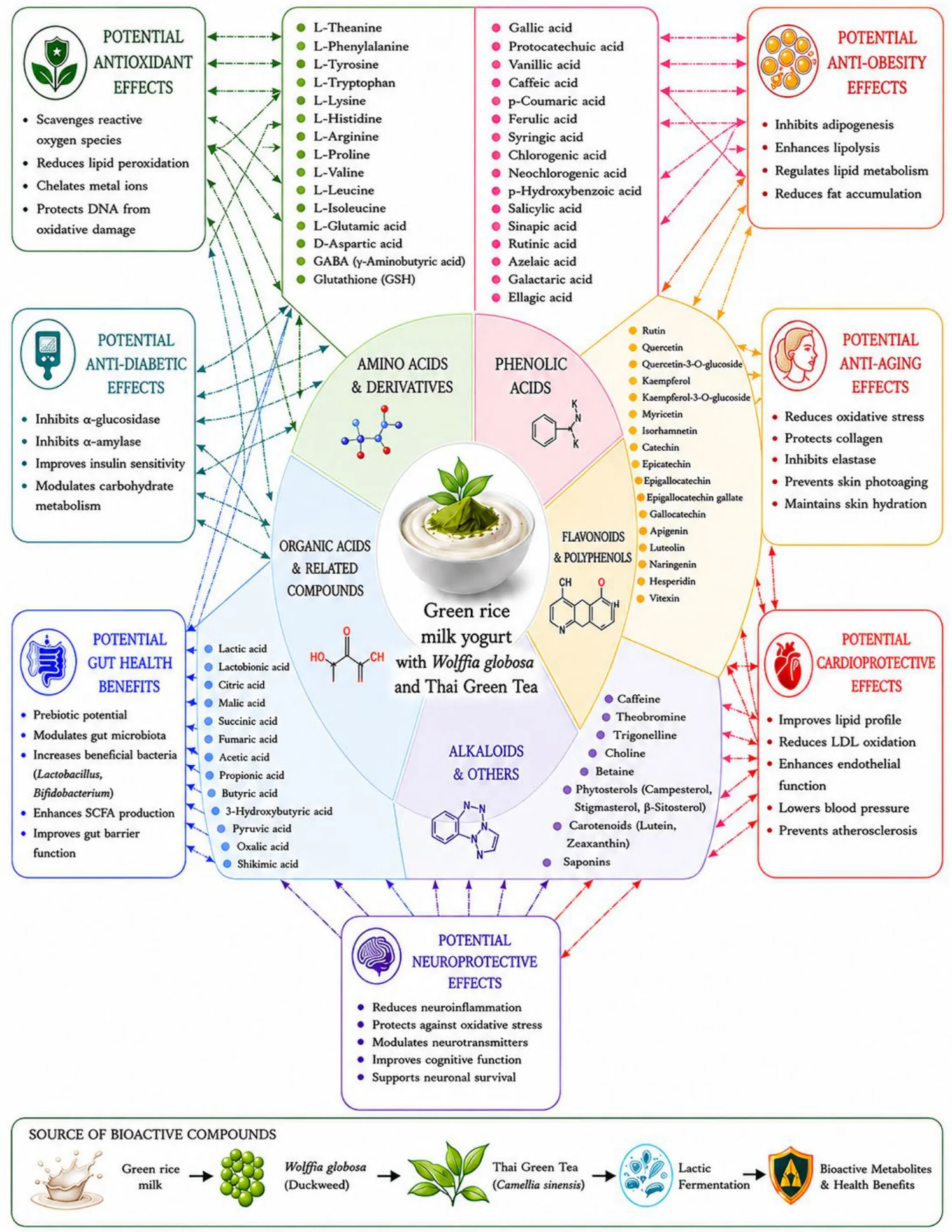
Conceptual bioactive metabolite network of green rice milk yogurt enriched with *W. globosa* and Thai green tea based on putatively annotated metabolites identified by UHPLC–QTOF–MS/MS and supported by the published literature. The diagram illustrates potential functional relationships and should be interpreted as a conceptual representation rather than direct experimental evidence.

Following Section 3.3, in particular Table S2, the current results of the UPLC–MS/MS metabolomic profiling of GRMY-WGP and that infused with Thai green tea revealed a diverse and interconnected bioactive metabolite network comprising amino acids and derivatives, phenolic acids, flavonoids and polyphenols, organic acids, alkaloids, phytosterols, carotenoids, and fermentation-derived metabolites. These compounds collectively contribute to the multifunctional health-promoting properties of the product through synergistic modulation of antioxidants, anti-inflammatory, antidiabetic, cardioprotective, neuroprotective, gut health, anti-aging, and anti-obesity pathways, as represented in Figure 2. The bioactive metabolites network map obtained from GRMY-WGP contains six metabolites including the following:

1. Amino acids and derivatives

The amino acid fraction included L-theanine, L-phenylalanine, L-tyrosine, L-tryptophan, L-lysine, L-histidine, L-arginine, L-proline, branched-chain amino acids (BCAAs), D-aspartic acid, γ-aminobutyric acid (GABA), and glutathione (GSH), representing a major functional component generated through fermentation and protein biotransformation. Based on the biological significance, L-theanine is a characteristic tea-derived amino acid associated with cognitive enhancement, stress reduction, and neuroprotection through modulation of neurotransmitter pathways. GABA contributes to neurophysiological regulation and may promote relaxation and neuronal protection. Glutathione (GSH) functions as a master intracellular antioxidant, protecting cells against oxidative stress. L-Arginine serves as a precursor for nitric oxide synthesis, supporting endothelial function and cardiovascular health. L-Tryptophan participates in serotonin biosynthesis and gut–brain axis regulation. In addition, the functional contributions of these metabolites are strongly associated with neuroprotective activity, antioxidant defense, cardiovascular protection, and metabolic regulation.

2. Phenolic acids

The phenolic acid group included gallic acid, protocatechuic acid, vanillic acid, caffeic acid, p-coumaric acid, ferulic acid, chlorogenic acid, neochlorogenic acid, salicylic acid, syringic acid, sinapic acid, ellagic acid, rutinic acid derivatives, and azelaic acid. The biological significance was exhibited by the phenolic acids, which are potent phytochemicals known to scavenge reactive oxygen species (ROS), inhibit lipid peroxidation, regulate inflammatory signaling pathways, and improve glucose metabolism. Among these compounds, gallic acid exhibited strong antioxidant and anti-inflammatory effects. Caffeic acid and chlorogenic acid are recognized for antidiabetic and anti-obesity activities. Ferulic acid contributed to vascular protection and oxidative stress reduction. Ellagic acid demonstrated chemopreventive and anti-aging properties. The functional contributions of phenolic acids, including AA, support anti-inflammatory responses, antidiabetic effects, and cardiometabolic health.

3. Flavonoids and polyphenols

The flavonoid-rich fraction is one of the most biologically important groups detected in the metabolomic profile. Key compounds included saponins, rutin, quercetin, quercetin-3-O-glucoside, kaempferol, kaempferol-3-O-glucoside, myricetin, isorhamnetin, catechin, epicatechin, epigallocatechin, epigallocatechin gallate (EGCG), gallocatechin, apigenin, luteolin, naringenin, hesperidin, vitexin, and galactaric acid. In terms of biological significance, tea-derived catechins and flavanols are recognized as highly potent antioxidants. For example, gallocatechin, EGCG, rutin, quercetin and kaempferol derivatives exhibit a strong free radical scavenging capacity, the suppression of the flavonoid-rich fraction enhances insulin sensitivity and protects against metabolic disorders.

Galactaric acid has been reported as an organic acid associated with antioxidant-related metabolic pathways, whereas saponins are recognized for their diverse biological activities, including antioxidant and anti-inflammatory properties. Although these metabolites were putatively annotated in the present study, their proposed biological roles were interpreted based on the previously published literature and should be considered as literature-supported hypotheses pending further experimental validation.

4. Organic acids and fermentation-derived metabolites

The fermentation process generated numerous organic acids, including lactic acid, lactobionic acid, citric acid, malic acid, succinic acid, fumaric acid, acetic acid, propionic acid, butyric acid, pyruvic acid, and shikimic acid. The biological significance of these metabolites indicated their role in active microbial metabolism or prebiotic effects (e.g., lactobionic acid), as well as their contributions to product quality (e.g., butyric acid and lactic acid) and physiological functionality, which encompasses gut health promotion, prebiotic activity, metabolic regulation, and fermentation quality.

5. Alkaloids and related bioactive compounds

This category included caffeine, theobromine, trigonelline, choline, and betaine. These compounds showed biological significance and functional contribution, in particular, caffeine, trigonelline, choline, and betaine, providing central nervous system stimulation, improved alertness, enhanced cognitive performance, glucose regulation, liver health, and antidiabetic activity.

6. Phytosterols and carotenoids

This metabolomic analysis also detected phytosterols, campesterol, stig-masterol, β-sitosterol, carotenoids, lutein, and zeaxanthin. Biological significance was recognized for cholesterol-lowering effects, cardiovascular protection, and anti-inflammatory properties and functional contributions associated with eye health, retinal protection, antioxidant defense, cardioprotective effects, anti-aging activity, and vision protection.

### 3.5. Metabolic Pathway Network of Bioactive Metabolites in GRMY-WGP

**Figure 3:**
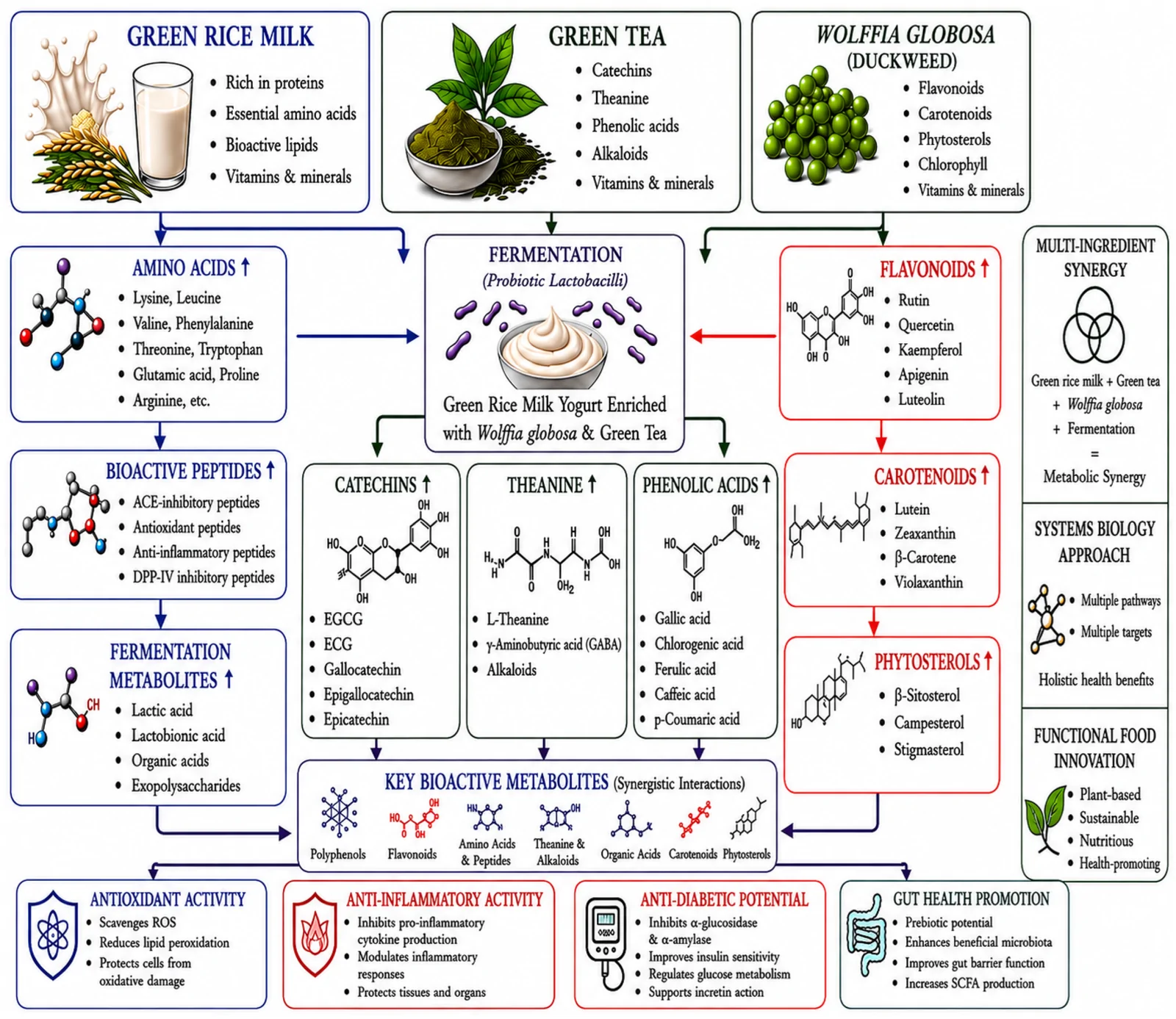
Conceptual metabolic pathway network illustrating the potential biological relationships among putatively annotated metabolites identified in green rice milk yogurt enriched with *W. globosa*. The proposed interactions are based on the literature interpretation and are presented as a conceptual hypothesis.

Figure 2 shows the results from the UPLC–MS/MS metabolomic category analysis, which revealed a complex and highly interconnected metabolic pathway network in GRMY-WGP, as shown in Figure 3. The identified metabolites were distributed into five major biochemical classes: (i) amino acids and derivatives, (ii) phenolic acids, (iii) flavonoids and polyphenols, (iv) organic acids and fermentation-derived compounds, and (v) alkaloids and related phytochemicals. These metabolite groups collectively contributed to the functional characteristics of the product through synergistic interactions across antioxidant, anti-inflammatory, antidiabetic, neuroprotective, cardio-protective, anti-obesity, anti-aging, and gut health pathways, including the following:

1. Amino acids and derivatives: Central regulators of cellular metabolism and neuroprotection

The amino acids include L-theanine, GABA, glutathione, and branched-chain amino acids, which serve as crucial intermediates in cellular metabolism and neuroprotection. L-theanine enhances cognitive function and stress reduction. GABA acts as an inhibitory neurotransmitter, aiding neuronal stability. Glutathione is a key antioxidant that protects against oxidative stress. L-arginine aids cardiovascular health through nitric oxide production. These amino acids bridge fermentation-induced proteolysis with neuroprotective and cardiometabolic pathways.

2. Phenolic acids: Core antioxidant and anti-inflammatory components

Phenolic acids, including gallic, caffeic, and ellagic acids, are significant for their antioxidant and anti-inflammatory properties. Originating from plant sources, these metabolites play a key role in cellular protection by regulating oxidative stress and metabolic functions. Gallic acid is noted for its potent antioxidant effects, while caffeic and chlorogenic acids help manage glucose and support anti-obesity actions. Ferulic acid contributes to vascular health, and ellagic acid is recognized for its anti-aging properties. Overall, phenolic acids are vital in modulating redox balance and inflammatory pathways.

3. Flavonoids and polyphenols: Principal bioactive hub of the network

Flavonoids and polyphenols are key bioactive compounds, with the flavonoid-rich fraction being the most biologically active. Major metabolites included rutin, quercetin, and EGCG, among others. Gallocatechin shows a strong AA, while rutin has multiple health benefits, including improved endothelial function. Quercetin and kaempferol derivatives enhance insulin sensitivity and protect against oxidative stress. EGCG modulates inflammatory and metabolic pathways. Overall, flavonoids serve as a vital bioactive hub that connects various health benefits such as antioxidant, anti-inflammatory, and cardioprotective mechanisms.

4. Organic acids and fermentation-derived metabolites: Drivers of gut health and metabolic regulation

Organic acids, including lactic, lactobionic, citric, malic, succinic, fumaric, acetic, propionic, butyric, 3-hydroxybutyric, pyruvic, oxalic, and shikimic acids, are primarily produced through microbial fermentation. Lactobionic acid improves mineral bioavailability and possesses antioxidant and prebiotic properties. Butyric acid is essential for maintaining intestinal barrier integrity, regulating inflammation, and supporting beneficial gut microbiota. Propionic and acetic acids, as short-chain fatty acids, impact glucose and lipid metabolism. Lactic acid enhances product preservation and probiotic stability. These organic acids are crucial in linking fermentation to gut health, metabolic regulation, and host-microbiome interactions.

5. Alkaloids and related phytochemicals: Neurocognitive and metabolic modulators

The alkaloid-related cluster included compounds such as caffeine, theobromine, trigonelline, choline, betaine, phytosterols, and carotenoids like lutein and zeaxanthin. Key roles included caffeine enhancing alertness and cognitive function while offering neuroprotection, theobromine supporting cardiovascular health, trigonelline aiding in neuroprotection and glucose regulation, choline and betaine participating in methylation and supporting neurological and liver functions, phytosterols reducing cholesterol absorption and improving cardiovascular health, and lutein and zeaxanthin protecting retinal tissues and acting as antioxidants. This group of metabolites is significant for linking neuroprotection, cardioprotection, and metabolic health.

### 3.6. Study Limitations

Although this study demonstrated the nutritional improvement, antioxidant activity, enzyme inhibitory activity, and metabolomic profile of GRMY-WGP samples, several limitations should be acknowledged. First, the proximate composition did not include moisture and ash contents, because these parameters were not determined during the compositional analysis. Consequently, the proximate composition presented in Table 1 does not represent a complete mass balance on a dry weight basis. Second, fermentation kinetics, including changes in pH, titratable acidity, and viable lactic acid bacteria throughout fermentation, were not monitored in the present study. Therefore, only the final fermentation characteristics are reported. Third, the sensory quality, consumer acceptability, color preference, flavor attributes, and storage stability were beyond the scope of the present investigation and should be evaluated in future studies to support commercial application. Fourth, the biological activities reported in this study were evaluated using in vitro assays. Therefore, further in vivo investigations and clinical validation are required before any health-promoting effects can be confirmed. Finally, the experimental design was based on three replicate measurements (*n* = 3), which represents the minimum number of observations for statistical analysis. Future studies should include larger biological sample sizes and independent fermentation batches to further improve the robustness of the findings.

Additionally, a limitation of this present metabolomic analysis is that it was performed by an external analytical facility, and only the processed metabolite annotation results were available to the authors. Consequently, detailed analytical quality control information, including pooled QC samples, internal standards, signal drift correction, and batch effect assessment, could not be independently verified. Future studies should incorporate a complete QA/QC workflow in accordance with current metabolomics reporting guidelines.

## 4. Conclusions

Overall, this study demonstrated that probiotic fermentation of green rice milk yogurt enriched with *W. globosa* powder (GRMY-WGP) and Thai green tea significantly enhanced its nutritional and functional properties through the generation of a diverse bioactive metabolite profile. Untargeted UHPLC–MS/MS metabolomic analysis revealed the presence of multiple classes of bioactive compounds, including amino acids, phenolic acids, flavonoids, organic acids, alkaloids, phytosterols, carotenoids, and fermentation-derived metabolites, which collectively contributed to the proposed bioactive metabolite network. Among the tested formulations, GRMY supplemented with 15% *W. globosa* powder (F4) exhibited the most favorable nutritional composition together with the richest metabolite profile, supporting its superior antioxidant capacity and enzyme inhibitory activities determined by in vitro assays. The identified metabolites suggest synergistic interactions among fermentation-derived metabolites, *Wolffia*-derived phytochemicals, and Thai green tea polyphenols, which may contribute to interconnected metabolic pathways associated with antioxidant defense, anti-inflammatory responses, glucose metabolism, and gut health promotion. Although the proposed bioactive metabolite network provides valuable mechanistic insight into the multifunctional potential of GRMY-WGP, these biological functions are inferred from metabolomic profiling together with in vitro bioactivity assays and should therefore be confirmed by targeted metabolomics, authentic compound validation, and in vivo studies. Nevertheless, the present findings provide a comprehensive metabolomic basis for the development of GRMY-WGP as a promising next-generation plant-based functional food and future food.

## Figures and Tables

**Table 1:** Nutritive values of green rice milk yogurt supplemented by *W. globosa* powder (GRMY-WGP) hydrolysates at different levels.

Assess	GRMY-WGP Formula			
	F1	F2	F3	F4
**Nutritive values (%, DW)**				
Protein content	16.68 ± 0.12 ^c^	21.14 ± 0.14 ^b^	21.14 ± 0.55 ^b^	22.48 ± 0.29 ^a^
Starch content	10.58 ± 0.32 ^c^	25.02 ± 0.50 ^b^	25.15 ± 0.53 ^b^	26.55 ± 0.52 ^a^
Dietary fiber content	7.59 ± 0.47 ^d^	25.30 ± 0.17 ^c^	35.38 ± 0.22 ^b^	42.45 ± 0.18 ^a^
Fat content	0.38 ± 0.22 ^b^	2.11 ± 0.13 ^a^	2.12 ± 0.05 ^a^	2.29 ± 0.02 ^a^

Note: All data are expressed as mean ± SD (*n* = 3). ^a–d^ Different letters in the same column indicate a significant difference (*p* < 0.05). Moisture and ash contents were not determined in the present study. Therefore, proximate composition values do not sum to 100%.

**Table 2:** Biological compounds and their activity in GRMY-WGP hydrolysates.

Assess	GRMY-WGP Hydrolysates			
	F1	F2	F3	F4
**Phytochemical compounds**				
TPC (mg GAE/g)	22.06 ± 1.43 ^d^	47.65 ± 1.95 ^c^	52.09 ± 0.68 ^b^	52.99 ± 0.50 ^a^
TFC (mg QUE/g)	24.97 ± 1.14 ^d^	54.45 ± 0.20 ^c^	59.45 ± 0.79 ^b^	60.45 ± 0.56 ^a^
DPPH activity (mg TROE/g)	3.82 ± 0.23 ^d^	4.18 ± 0.41 ^c^	5.21 ± 0.45 ^b^	5.48 ± 0.45 ^a^
ABTS activity (mg TROE/g)	340.29 ± 0.98 ^d^	348.31 ± 1.43 ^c^	378.91 ± 1.03 ^b^	424.15 ± 2.67 ^a^
FRAP capacity (mg TROE/g)	29.18 ± 2.45 ^d^	51.58 ± 2.20 ^c^	62.67 ± 4.46 ^b^	106.35 ± 1.61 ^a^
β-carotene (mg/g)	0.69 ± 0.04 ^c^	0.93 ± 0.04 ^b^	0.95 ± 0.03 ^b^	1.81 ± 0.10 ^a^
**Antidiabetic activities**				
α–glucosidase inhibition (IC_50_, mg/mL)	1.73 ± 0.35 ^b^	1.53 ± 0.35 ^a,b^	1.47 ± 0.35 ^a,b^	1.41 ± 0.39 ^a^

Note: All data are expressed as the mean ± SD (*n* = 3). ^a–d^ Different letters within the same column indicate a significant difference (*p* < 0.05).

## Data Availability

Data are contained within the article.

## Supplementary Materials

The following supporting information can be downloaded at https://www.mdpi.com/article/10.3390/life16081385/s1, Figure S1: UPLC–QTOF based peak chromatogram of GRMY-WGP hydrolysates for F1, control. (A) ESI negative mode and (B) ESI positive mode; Figure S2: UPLC–QTOF based peak chromatogram of GRMY-WGP hydrolysates for F4, GRMY-WGP supplemented 15% *Wolffia globosa*. (A) ESI negative mode and (B) ESI positive mode; Table S1: The comparative results of eighty-eight compounds obtained from LC-QTOF analysis revealed their pharmacological and biological effects when explored in both negative and positive modes; Table S2: Representative metabolite annotations selected from the 88 putatively annotated metabolites for functional classification and biological interpretation.

## Abbreviations

The following abbreviations are used in this manuscript:

GRM	Green rice milk
WGP	*Wolffia globosa* powder
GRMY-WGP	Green rice milk yogurt enhanced with *Wolffia globosa* powder
RBRMY-WGP	Riceberry rice milk yogurt supplemented with *Wolffia globosa* powder
AA	Antioxidant activity
DPPH assay	2,2-diphenyl-1-picrylhydrazyl
ABTS assay	2,2-azino-bis-3-ethylbenzothiazoline-6-sulphonic acid
FRAP assay	Ferric reducing antioxidant power
TPC	Total phenolic content
TFC	Total flavonoid content
GAE	Gallic acid equivalent
QUE	Quercetin equivalent
TROE	Trolox equivalent
IC_50_	Half-maximal inhibitory concentration
LC-MS/MS	Liquid chromatography–tandem mass spectrometry
LC-QTOF	Liquid chromatography–quadrupole time-of-flight mass spectrometry
UPLC-QTOF	Ultra-performance liquid chromatography–quadrupole time-of-flight mass spectrometry
LC-ESI-MS/MS	Liquid chromatography- electrospray ionization–tandem mass spectrometry
GC-MS/MS	Gas chromatography–tandem mass spectrometry
