# Supplementary material for: Untargeted Metabolomics Reveals Functional Bioactive Metabolite Networks in Green Rice Milk Yogurt Enriched with Wolffia globosa
Source: Life (Basel). 2026 Aug 21;16(8):1385. doi: 10.3390/life16081385 (PMC13514272; doi:10.3390/life16081385)
Supplement: Supplementary file 1 [file life-16-01385-s001.zip › life-4458075-supplementary.pdf]

Supplementary data

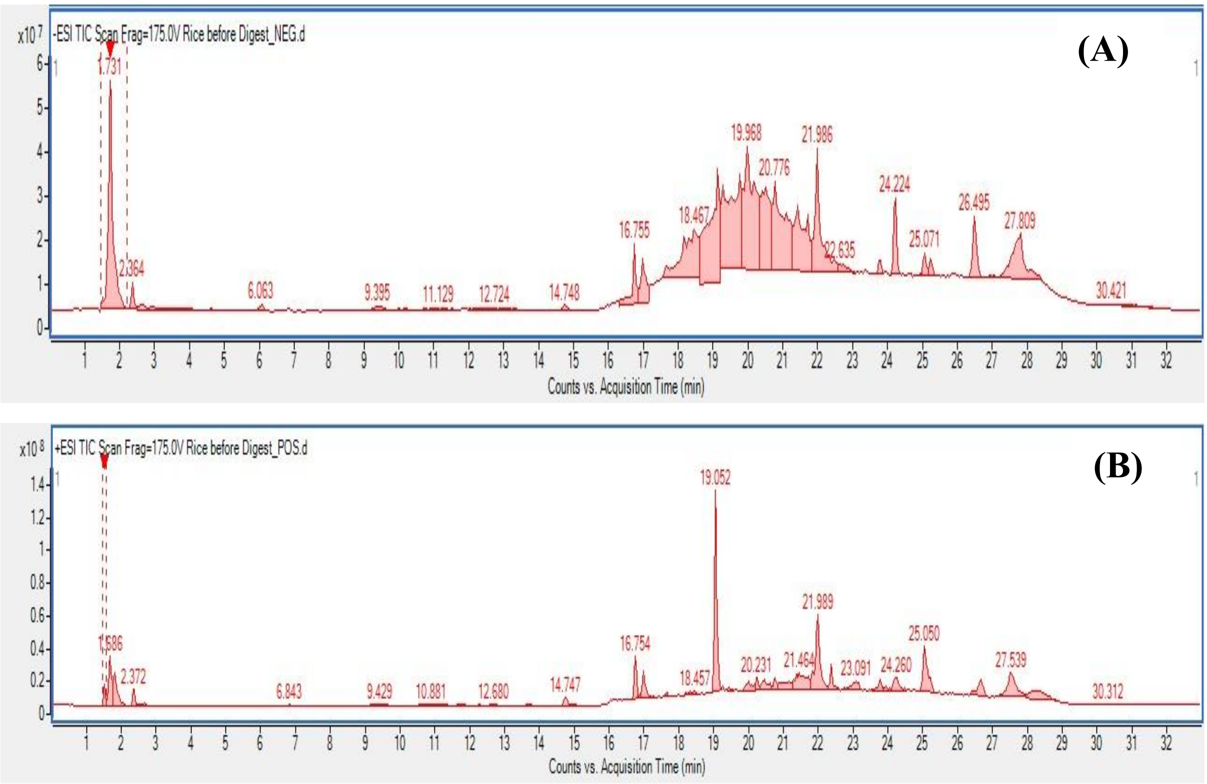

Figure S1. UPLC–QTOF based peak chromatogram of GRMY-WGP hydrolysates for F1; control. (A) ESI negative mode and (B) ESI positive mode.

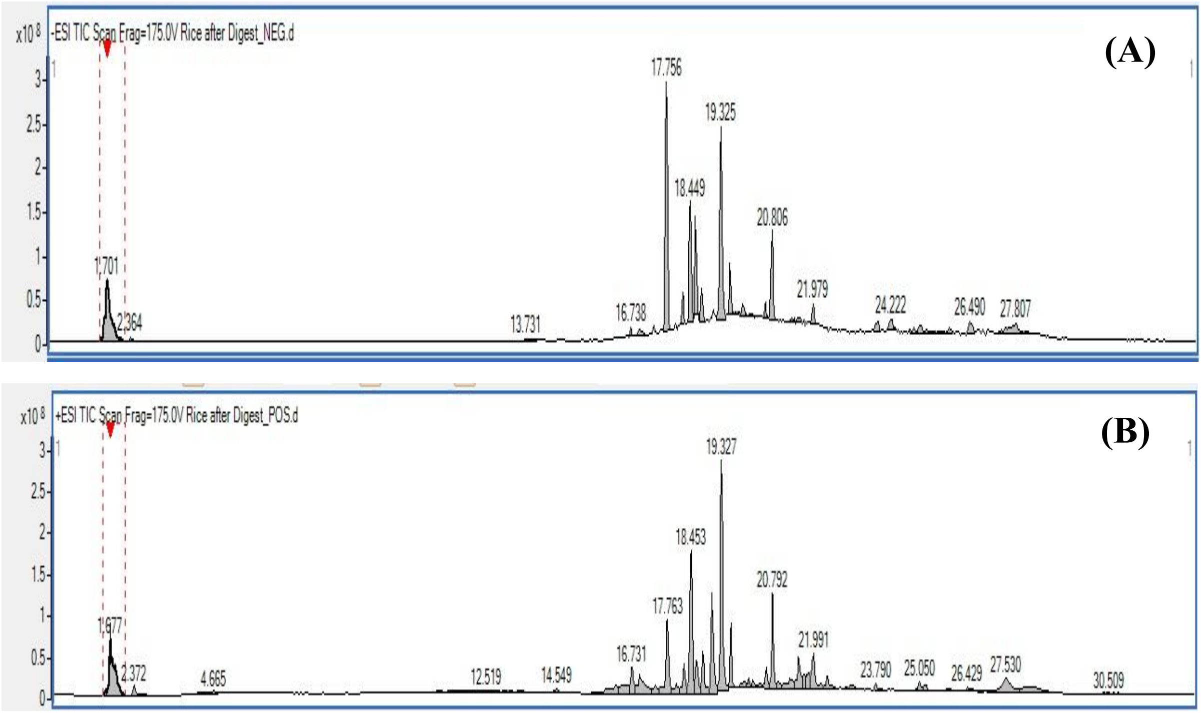

Figure S2. UPLC–QTOF based peak chromatogram of GRMY-WGP hydrolysates for F4; GRMY-WGP supplemented 15% *Wolffia globosa*. (A) ESI negative mode and (B) ESI positive mode.

.

Table S1. The comparative results of eighty-eight compounds obtained from LC-QTOF analysis revealed their pharmacological and biological effects when explored in both negative and positive modes.

**No.**	**Compounds**	**ESI Mode**	**RT**	***m/z***
1	Leucodelphinidin 3-O-α-L-rhamnopyranoside	+	1.728	513.1225
2	Gossypetin 8-glucoside-3-sulfate	+	1.77	559.0432
3	Chondroitin 6-sulfate	+	1.966	917.1179
4	Taurine	+	19.689	482.2958
5	Piperacillin β-lactam	+	19.789	516.6100
6	Crizotinib	+	1.76	494.1197
7	Duloxetine	+	1.978	342.1137
8	Lappaol A	+	9.894	581.2037
9	Isoneotheaflavin	+	17.061	563.1198
10	Macaflavone II	+	17.880	469.1392
12	Ophiopogonin C	+	16.73	853.4612
13	Licoricesaponin G2	+	16.492	837.392
14	Acarbose	+	17.126	644.2431
15	Ergotamine	+	17.421	580.2566
16	Azelaic acid	+	17.178	187.0976
17	Tauroursocholic acid	+	17.011	514.2845
18	Glycocholic acid	+	18.114	464.3025
19	Prostaglandin E2 p-acetamidophenyl ester	+	17.287	530.2794
20	Vignatic acid B	+	17.735	518.2916
21	Glimepiride	+	18.065	535.2239
22	Cerivastatin	+	18.169	504.2423
23	Lucidenic acid B	+	18.803	473.2522
24	Ganolucidic acid A	+	19.157	545.3156
25	Dihydroresveratrol	+	19.422	229.0876
26	Chrysophanol 8-(6-galloylglucoside)	+	19.351	567.113
27	Isobutyl salicylate	+	19.479	193.0872
28	Embelin	+	20.1	293.1797
29	Myrsinone	+	19.841	293.1762
30	Glutathione Cobalamin	+	1.627	816.8202
31	Histidinyl-Cysteine	+	18.28	303.0771
32	Docusate	+	19.311	421.2275
33	Tibolone	+	22.209	311.2017
34	Vitamin D derivatives (6RS / 6R	+	20.919	467.3015
35	Armillarivin	+	21.149	383.1849
36	Fasciculic acid A	+	21.518	619.4209
37	Myricetin 3,7-diglucuronide	+	24.139	669.0845
38	Lisuride	+	26.521	337.2057
39	Tabernamine	+	21.811	661.3841
40	Propranolol	+	22.208	258.1502
41	Methacycline	+	27.551	441.1273
42	Leucomycin A8	+	20.927	391.7104
43	Fosinopril	+	18.425	578.2932
44	BAY-60-7550	+	19.964	521.2439
45	Resveratrol 4'-(2-galloylglucoside)	-	1.881	541.1319
46	Epicatechin 3-O-(4-methylgallate)	-	1.798	455.1013
47	Hesperetin 7-O-glucoside	-	1.911	509.1351
48	Malvidin 3-(6-malonylglucoside) 5-glucoside	-	1.767	741.1829
49	Nateglinide	-	10.179	316.1918
50	Cabergoline	-	18.162	450.2864
51	Pergolide sulfone	-	6.272	391.1698
52	Mycalamide A	-	16.779	548.2696
53	Ophiopogonin D	-	16.747	853.4577
54	Elatoside E	-	16.445	881.4759

55	1-O-Sinapoylglucose	-	1.739	385.1179
56	3-Aminopropanesulphonic acid	-	1.721	138.0248
57	N4-Phosphoagmatine	-	16.985	209.0796
58	Sertraline carbamic acid glucuronide	-	1.7	524.0882
59	Crizotinib	-	1.748	494.1205
60	Retapamulin	-	17.16	562.3226
61	Estazolam	-	17.634	293.0635
62	Acitretin	-	18.049	341.1717
63	Triamcinolone hexacetonide	-	16.623	531.2794
64	Latanoprost ethyl amide (Latanoprost Analog)	-	17.572	462.2853
65	Tafluprost ethyl amide (prostaglandin derivative)	-	1.807	482.2343
66	Capreomycin	-	14.216	667.343
67	Premithramycin A2'	-	1.701	719.2079
68	Tetracenomycin F2	-	1.747	383.0753
69	N-Oleoyl Dopamine	-	19.573	462.3204
70	Mangostenone B	-	19.273	507.2044
71	Verapamil	-	19.322	453.2845
72	Fluphenazine enanthate	-	18.475	594.2614
73	Clarithromycin	-	19.583	746.4655
74	Indoramin	-	20.802	392.2023
75	Rifaximin	-	20.804	391.6761
76	Sufentanil	-	21.373	431.2021
77	Ciclesonide	-	19.447	539.3047
78	Methazolamide	-	19.111	235.0001
79	4-Ketolutein F	-	18.224	581.4049
80	3,5-Dihydroxyphenyl 1-O-(6-O-galloyl-beta-D-glucopyranoside)	-	1.726	439.0894
81	Squamosinin A	-	27.265	621.4368
82	Taraxacolide 1-O-b-D-glucopyranoside	-	24.215	427.1989
83	Ecabet	-	23.823	379.1591
84	Quinagolide		26.506	440.2219
85	Ramelteon	-	22.062	258.149
86	Jurubine	-	25.06	594.3948
87	Brosimacutin C	-	24.196	341.1385
88	9(S)-HpOTrE	-	21.985	309.2065

Table S2. Representative metabolite annotations selected from the 88 putatively annotated metabolites for functional classification and biological interpretation.

No.	Compound	Metabolite Class	Associated Pathway	Biological Activity	Reported Source	Scientific Evidence Summary
1	Lactobionic acid	Organic acid / Fermentation metabolite	Lactose oxidation and microbial fermentation pathway	Prebiotic, antioxidant, mineral absorption enhancement	Fermented dairy products and probiotic fermentation systems	Reported to improve gut microbiota balance and antioxidant properties in fermented foods
2	Theophylline	Methylxanthine alkaloid	Purine alkaloid metabolism	Neurostimulatory, bronchodilatory, antioxidant	Tea leaves including green tea	Widely reported as a bioactive alkaloid in tea beverages
3	D-Aspartic acid	Amino acid	Amino acid metabolism	Neuroprotective, metabolic regulation	Fermented soybean and microbial metabolism	Associated with protein hydrolysis during fermentation
4	Taurine	Sulfonic amino acid	Sulfur amino acid metabolism	Antioxidant, anti-inflammatory, osmoregulatory	Microbial fermentation and functional foods	Known for membrane protection and antioxidant effects
5	Quinic acid	Phenolic acid derivative	Shikimate pathway	Antioxidant, anti-inflammatory	Tea leaves and plant phenolics	Frequently identified in tea metabolomics studies
6	(+)-Gallocatechin	Catechin / Flavonoid	Flavonoid biosynthesis pathway	Strong antioxidant, anti-diabetic, anti-inflammatory	Green tea and Camellia sinensis products	Major catechin group reported in green tea
7	4-O-Caffeoylshikimic acid	Phenolic acid	Phenylpropanoid metabolism	Antioxidant, ROS scavenging	Tea and plant-derived beverages	Phenolic acids are associated with oxidative stress reduction
8	Luteone 7-glucoside	Flavonoid glycoside	Isoflavonoid biosynthesis	Antioxidant, phytoestrogenic activity	Soybean and legume metabolites	Isoflavonoids are common functional compounds in soy products
9	Viniferifuran	Stilbenoid polyphenol	Phenylpropanoid pathway	Antioxidant, antimicrobial	Plant polyphenol metabolite	Polyphenols linked with oxidative stress reduction
10	beta-penta-O-Galloyl-glucose	Hydrolyzable tannin	Gallate and polyphenol metabolism	Strong antioxidant, anti-cancer, anti-inflammatory	Tea and medicinal plants	Galloyl compounds are associated with radical scavenging activity
11	Dihydrocaffeic acid 3-O-glucuronide	Phenolic metabolite	Phenylpropanoid metabolism	Antioxidant and anti-inflammatory	Tea polyphenol metabolism	Common metabolite associated with phenolic biotransformation
12	Purine	Nitrogen-containing heterocycle	Purine metabolism	Cellular energy regulation	Microbial and plant metabolism	Detected in fermented functional foods
13	Hypoxanthine	Purine derivative	Nucleotide degradation pathway	Energy metabolism biomarker	Fermented foods and microbial metabolism	Associated with ATP degradation and fermentation metabolism

14	3-Fucosyllactose	Oligosaccharide	Carbohydrate metabolism	Prebiotic and gut microbiota modulation	Fermented dairy and functional foods	Known to stimulate beneficial gut bacteria
15	Ascorbalamic acid	Vitamin C derivative	Ascorbate metabolism	Antioxidant	Fermented plant products	Related to oxidative protection systems
16	Rotenonone	Rotenoid compound	Secondary metabolite pathway	Antioxidant and bioactive phytochemical activity	Plant-derived metabolites	Rotenoids possess diverse biological activities
17	Gossypetin 8-glucoside-3-sulfate	Flavonol glycoside	Flavonoid biosynthesis	Antioxidant, anti-inflammatory	Plant polyphenol metabolite	Flavonol derivatives are potent ROS scavengers
18	Cornuside I	Iridoid glycoside	Terpenoid biosynthesis	Anti-inflammatory and neuroprotective	Medicinal plant metabolite	Iridoids are associated with anti-inflammatory effects
19	Fructoselysine 6-phosphate	Maillard-related metabolite	Amino sugar metabolism	Microbial nutrient metabolism	Fermented foods	Associated with microbial fermentation and amino acid transformation
20	6-O-Glucosylmaltose	Oligosaccharide	Carbohydrate metabolism	Prebiotic potential	Fermented carbohydrate-rich foods	Oligosaccharides support probiotic bacteria
21	Epigallocatechin gallate (EGCG)	Catechin / Polyphenol	Flavonoid biosynthesis	Potent antioxidant, anti-inflammatory, anti-diabetic	Thai green tea (Camellia sinensis)	EGCG is widely recognized as the major bioactive catechin in green tea with strong ROS scavenging capacity
22	Genistein	Isoflavone	Isoflavonoid biosynthesis	Phytoestrogenic, antioxidant, anti-cancer	Soybean-derived metabolite	Genistein is a major soy isoflavone associated with cardiovascular and metabolic health benefits
23	Daidzein	Isoflavone	Phenylpropanoid and isoflavonoid metabolism	Antioxidant, anti-inflammatory	Soybean and fermented soy foods	Daidzein is frequently enriched during soy fermentation due to glycoside hydrolysis
24	Catechin	Flavan-3-ol	Flavonoid biosynthesis	Antioxidant, anti-obesity, cardioprotective	Green tea and tea beverages	Catechin compounds exhibit strong free radical scavenging properties
25	Gallic acid	Phenolic acid	Phenylpropanoid metabolism	Antioxidant, antimicrobial	Tea polyphenols and fermented plant products	Gallic acid is associated with oxidative stress reduction and antimicrobial activity
26	Lactic acid	Organic acid	Fermentation metabolism	Antimicrobial and probiotic-associated metabolite	Lactic acid bacteria fermentation	Major metabolite produced during yogurt fermentation
27	GABA (Gamma-aminobutyric acid)	Bioactive amino acid	Glutamate metabolism	Neuroprotective, anti-hypertensive	Fermented soybean products	GABA accumulation is commonly enhanced during lactic fermentation
28	Quercetin	Flavonol	Flavonoid biosynthesis	Antioxidant, anti-inflammatory	Tea and plant-derived metabolites	Quercetin derivatives are strong ROS scavengers
29	Caffeic acid	Hydroxycinnamic acid	Phenylpropanoid pathway	Antioxidant and antimicrobial	Tea and fermented plant foods	Phenolic acids contribute to antioxidant defense mechanisms
30	Kaempferol	Flavonoid	Flavonoid biosynthesis	Anti-inflammatory, antioxidant	Tea leaves and soy products	Kaempferol is associated with cellular protection against oxidative damage

31	Raffinose	Oligosaccharide	Carbohydrate metabolism	Prebiotic activity	Soybean carbohydrate fraction	Raffinose family oligosaccharides support probiotic bacteria growth
32	Succinic acid	Organic acid	TCA cycle	Energy metabolism biomarker	Fermentation metabolism	Succinic acid is commonly detected in microbial fermentation systems
33	Ferulic acid	Phenolic acid	Phenylpropanoid pathway	Antioxidant and anti-inflammatory	Tea and cereal-associated phenolics	Ferulic acid is a potent antioxidant compound
34	Theanine	Non-protein amino acid	Amino acid metabolism	Relaxation-promoting and neuroprotective	Green tea	Theanine is a unique amino acid associated with tea functionality
35	Pyrogallol	Phenolic compound	Polyphenol metabolism	Antioxidant and antimicrobial	Tea and fermented polyphenol products	Pyrogallol derivatives are potent reducing compounds
36	Mannose	Monosaccharide	Carbohydrate metabolism	Immune modulation and antioxidant support	Plant-derived carbohydrates	Mannose participates in glycoprotein biosynthesis and antioxidant pathways
37	Tryptophan	Essential amino acid	Amino acid metabolism	Precursor for serotonin and bioactive metabolites	Soy protein hydrolysis products	Fermentation may increase free amino acid availability
38	Phenylalanine	Aromatic amino acid	Phenylpropanoid precursor pathway	Precursor for phenolic biosynthesis	Soybean and fermentation metabolites	Phenylalanine is linked to biosynthesis of antioxidant phenolics
39	Gluconic acid	Sugar acid	Glucose oxidation pathway	Antioxidant support and acidification	Fermented foods and microbial metabolism	Gluconic acid contributes to acidity and oxidative metabolism
40	Ginsenoside-like triterpenoid	Triterpenoid	Secondary metabolite biosynthesis	Antioxidant and adaptogenic potential	Plant-derived phytochemical	Triterpenoids are associated with membrane protection and bioactivity
41	Epicatechin	Flavan-3-ol / Polyphenol	Flavonoid biosynthesis pathway	Antioxidant, cardioprotective, anti-inflammatory	Green tea and plant-derived foods	Epicatechin is a major tea flavonoid associated with strong free radical scavenging activity
42	Genistin	Isoflavone glycoside	Isoflavonoid biosynthesis	Phytoestrogenic, antioxidant	Soybean metabolites	Genistin is a major soybean isoflavone precursor commonly transformed during fermentation
43	Daidzin	Isoflavone glycoside	Phenylpropanoid and isoflavonoid metabolism	Antioxidant and anti-inflammatory	Soybean and fermented soy products	Fermentation can hydrolyze daidzin into more bioavailable aglycone forms
44	Epigallocatechin	Catechin / Flavonoid	Flavonoid metabolism	Potent antioxidant, anti-diabetic	Thai green tea	Epigallocatechin is one of the dominant antioxidant catechins in green tea
45	Chlorogenic acid	Phenolic acid	Phenylpropanoid pathway	Antioxidant, anti-obesity	Tea and plant-based beverages	Chlorogenic acid is associated with metabolic regulation and antioxidant activity
46	Pyruvic acid	Central carbon metabolite	Glycolysis and TCA cycle	Energy metabolism regulation	Microbial carbohydrate metabolism	Pyruvate acts as a central metabolite linking glycolysis and secondary metabolism
47	Quercetin glycoside	Flavonol glycoside	Flavonoid biosynthesis	Antioxidant, anti-inflammatory	Tea polyphenols	Quercetin derivatives possess strong ROS scavenging activity

Note: The "Source" column indicates the organism or food matrix in which the metabolite has been previously reported according to public metabolite databases. It does not represent the biological source or ingredient used in the present study.
